# A method to assess the quality of additive manufacturing metal powders using the triboelectric charging concept

**DOI:** 10.1038/s41598-024-67295-0

**Published:** 2024-07-16

**Authors:** E. Galindo, E. R. L. Espiritu, C. Gutierrez, Ali N. Alagha, P. Hudon, M. Brochu

**Affiliations:** Department of Mining and Materials Engineering, McGill University, 3610 University Street, Montreal, QC H3A 0C5 Canada

**Keywords:** Tribocharging phenomenon, Surface chemistry, Metallic powders, Feedstock quality, Materials science, Techniques and instrumentation

## Abstract

A new method to assess the quality of additive manufacturing (AM) metal powders using the triboelectric charging concept is demonstrated using CpTi, Ti6Al4V, AlSi10Mg, IN 738, and SS 316L powders. For each powder tested, the surface chemical composition was first analyzed using X-ray photoelectron spectroscopy (XPS) to determine the composition of the passivation layer. Some modifications to the current GranuCharge™ setup, developed by GranuTools™, were then performed by incorporating a flow rate measuring tool to assess how tribocharging is affected as a function of flow rate. Variations in the tribocharging response have been found with the flow rate of CpTi, AlSi10Mg and SS 316L powders. Moreover, results suggest that the tribocharging behavior might not be the same even with powders fabricated with the same passivation process. Finally, the compressed exponential model of Trachenko and Zaccone was used to reproduce the tribocharging behavior of the powders. The models were found to work best when the stretch constant *β* = 1.5, which is identical to the value found in other systems such as structural glasses, colloidal gels, entangled polymers, and supercooled liquids, which experience jamming when motion of individual particles become restricted, causing their motion to slow down.

## Introduction

In many powder bed additive manufacturing processes such as laser powder bed fusion and binder jetting, the quality of the spread layer, which is characterised by its continuity, uniformity, and packing density, must be maximised to reduce surface defects and porosity^3,4^. Several standard powder characterisation tools are used to assess powder properties. However, there is currently no single metric to evaluate the feedstock quality. Moreover, conventional characterization techniques often only quantify bulk properties, making it difficult to detect minor variations on powder surface.

Currently, the most employed surface-sensitive methods are Auger spectroscopy and X-ray photoelectron spectroscopy (XPS), but they are expensive, time-consuming, and require significant expertise and experienced users, which is not compatible with industrial manufacturing. Recently, however, a new user-friendly powder triboelectrometer^1^, which combines different triboelectrification mechanisms (particle–particle friction, particle–wall friction, and particle–wall impact), is now available to characterize surface properties. Tribocharging, which is also referred to as triboelectric charging or frictional electrification, is a surface phenomenon produced by the development of an electrostatic charge when two materials are put in contact or rubbed against each other and separated^5,6^. This can be accomplished using cascade, vibration, or fluidization methods. The new powder triboelectrometer^1^, mentioned above, uses the cascade method.

Charge transfer can occur via ion transfer, electron transfer, or mass transport^6–9^. In the case of metal–metal contact, tribocharging is produced by electron transfer^10^ and is believed to be driven by the work function, defined as the minimum energy required to remove an electron from a metal surface^9,10^. The work function theory states that when two materials are in contact, electron transfer occurs until their “conduction bands are filled to the same level and their Fermi levels equalize”^5,6,9^. For a metal–insulator contact and an insulator-insulator contact, the work function theory does not apply, since no “free electrons” occur at the surface of an insulator^9^. This can be explained by the effective work function theory^6,9^, which assumes that available electrons exist at the surface electronic level, not in the bulk (for a good introduction and a deeper understanding of the triboelectric charging phenomena, see the reviews of Matsuaka et al.^6^ and Mirkowska et al.^9^). Numerous empirical tribocharging studies with different pairs of materials have led to triboelectric series, which rank different materials in order of their likelihood in gaining a positive or negative charge following a “contact separation” process^5,11^. Unfortunately, many contradictory series have been published^5^ since each series was obtained empirically.

Given the concept that all materials have a defined work function using a reference material to generate the electron transfer, the tribocharging concept should be able to measure the surface characteristics of a powder such as the surface state of the material. Furthermore, charging models can be used to interpret the measurements obtained from the tribocharging process and yield quantitative characteristics proper to the nature of the powder surface. In this work, a new powder surface characterisation methodology based on the effective work function measurement is proposed. The surface chemical composition of the investigated powders was first analyzed using X-ray photoelectron spectroscopy (XPS) to determine the composition of the passivation layer. An existing tribocharging equipment was then modified to add data acquisition related to charging flow rate. Modified powder law models were used to analyse the time-dependent charging behavior and determine critical material-dependent tribocharging constants. The demonstration was performed on the main additive manufacturing (AM) powder families: Ti, Al, Fe, and Ni.

## Materials and methods

### Powder sourcing and characterization

**Figure 1:**
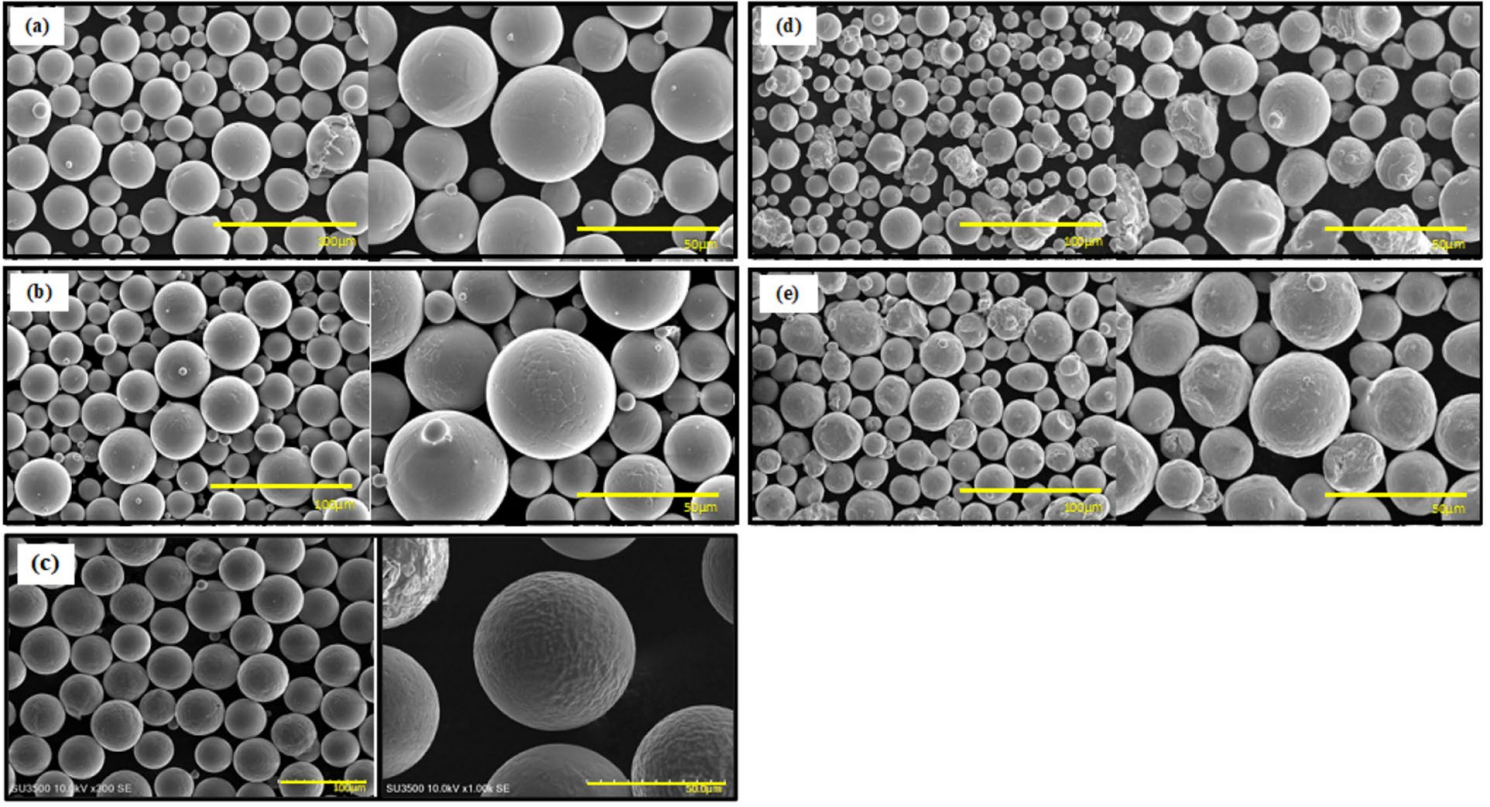
SEM micrographs of (**a**) CpTi, (**b**) Ti6Al4V, (**c**) AlSi10Mg, (**d**) IN 738 and (**e**) SS 316L powders.

A series of commercially available AM-grade powders were used. The alloys, their particle size distribution (PSD) represented by the three main diameters, specific surface area (SSA) (measured using a Microtrac MRB SYNC Particle Size Analyzer, Microtrac MRB, Japan), and their sources are presented in Table 1. The SSA was obtained from the PSD raw data, specifically, by dividing the surface area of each particle over the volume times the density of the specific powder, and finally taking the average of all particles. The powder morphology was determined using a Hitachi SU3500 Scanning Electron Microscope (SEM; Hitachi, Japan). The results are presented in Fig. 1. The micrographs show that CpTi, Ti6Al4V and AlSi10Mg powders are mostly spherical with a small number of irregular particles and particles with satellites. The IN 738 and SS 316L powders have spherical, irregular, elongated particles and some satellites. The side-by-side micrographic comparison of the powders also indicates that the particle sizes of the different alloys are similar.

**Table 1:** Sample particle size distribution expressed as D_10_, D_50_ and D_90_ (µm), and the calculated SSA (m^2^/g).

Alloy	D_10_	D_50_	D_90_	SSA	Source
CpTi (grade 1)	25	34	50	0.05	AP&C
Ti6Al4V (grade 23)	27	36	51	0.05	AP&C
AlSi10Mg	43	55	74	0.04	Equispheres
IN 738	21	32	49	0.05	Praxair Surface Technologies Inc
SS 316L	26	36	53	0.03	LPW Technology

### Powder surface characterisation using XPS

For each powder tested, the surface chemical composition was analyzed using a Thermo Scientific K-Alpha monochromatic X-ray photoelectron spectrometer (Thermo Fisher Scientific Inc., USA) equipped with an Al Kα X-ray source (1486.6 eV, 0.834 nm). Elemental survey (pass energy of 200 eV) and high-resolution scans (pass energy of 50 eV) were acquired in a high vacuum chamber at 10^–8^ Torr. An electron charge gun was employed to avoid surface charge effects. The spectra were calibrated using C 1 s peak for C–C (284.8 eV) and fitted using Avantage processing software (Thermo Fisher Scientific Inc., USA). The spectral fitting parameters were based on the NIST database^12^ and the curve-fitting procedures of Biesinger et al.^13^.

### Triboelectric charging trials

**Figure 2:**
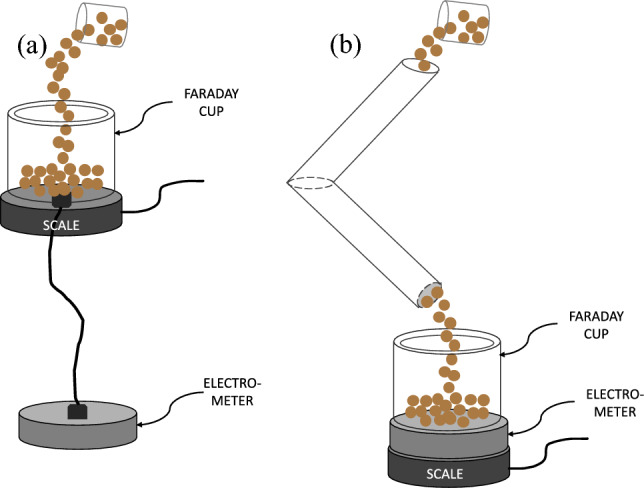
GranuCharge measurement of (**a**) initial charge and (**b**) final charge with modification.

The tribocharging campaigns were performed at 25 °C and 35% of relative humidity in a modified GranuCharge™ equipment (GranuTools™, Belgium), where instead of only measuring the charge as a function of time, a scale was positioned to measure the mass as a function of time, as seen in Fig. 2. This apparatus uses a V-tube geometry that combine the different mechanisms leading to triboelectrification: (1) friction between the grains, (2) friction between the grains and the wall, and (3) the impact of the grains on the wall at the connection between the two tubes^4^. The procedure was to first measure the time-dependent initial charge of the powder to correct the charge gain occurring during the process. The powder was then fed into the V-tubes at a constant flow rate and the time-dependent charging was recorded. The corrected charging rate was then plotted as a function of the flow rate and analysed. The tribocharge campaigns were done using stainless steel tubes and each powder lot was tested three times to assess the repeatability and reliability of the data.

## Results

### Surface chemistry/composition characterisation

#### CpTi powder

**Figure 3:**
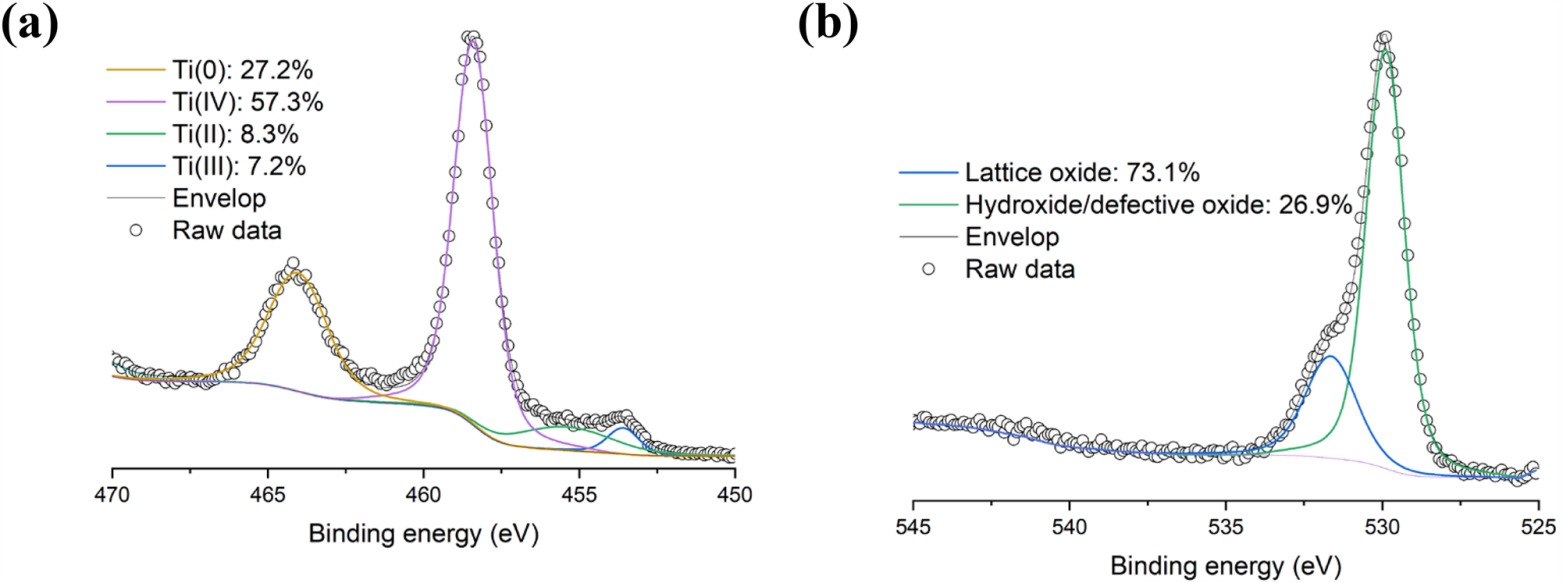
High-resolution (**a**) Ti 2p and (**b**) O 1s scans of CpTi powder.

XPS elemental survey of the CpTi sample shown in Table 2 indicated the presence of other elements such as C, N, and O at the surface. To determine the type of oxide at the surface, high-resolution scans of Ti 2p and O 1s were collected and deconvoluted into their individual components, as shown in Fig. 3. As depicted in Fig. 3a, for Ti 2p the dominant peak was found at 458.7 eV, which is assigned to Ti(IV) or TiO_2_. A peak at 453.9 eV, corresponding to Ti metal, was also fitted. Other peaks such as Ti(II) or TiO at 455.2 eV and Ti(III) or Ti_2_O_3_ at 457.3 eV were also detected during peak-fitting. These findings suggest that the oxide film at the surface of pure Ti powder consists of mixed oxides of TiO_2_, Ti_2_O_3_, and TiO after passivation and handling. This is consistent with observations reported previously^14–16^. Figure 3b depicts the deconvolution of the O 1s spectrum into lattice oxide (at 530.2 eV) and hydroxide/defective oxide (at 531.9 eV) yielded a relative composition of 73.1 and 26.9 at. %, respectively. Interestingly, the relative content attributed to lattice oxide (73.1 at. %) is close to the relative content of Ti oxides at the surface (72.8 at. %).

**Table 2:** Surface composition (in at. %) of CpTi powder measured by XPS.

Sample	Ti 2p	C 1s	N 1s	O 1s
CpTi	15.6	37.8	1.5	45.1

#### Ti6Al4V powder

Table 3 present the XPS elemental survey of the surface of the Ti6Al4V sample. In addition to Ti, Al, and O, the survey also identified C as a surface contaminant while V was not detected at the surface, which agrees with previous reports^16,17^.

**Table 3:** Surface composition (in at. %) of Ti6Al4V powder from XPS elemental survey.

Sample	Ti 2p	Al 2p	C 1s	N 1s	O 1s
Ti6Al4V	14.5	8.6	25.6	1.0	50.3

**Figure 4:**
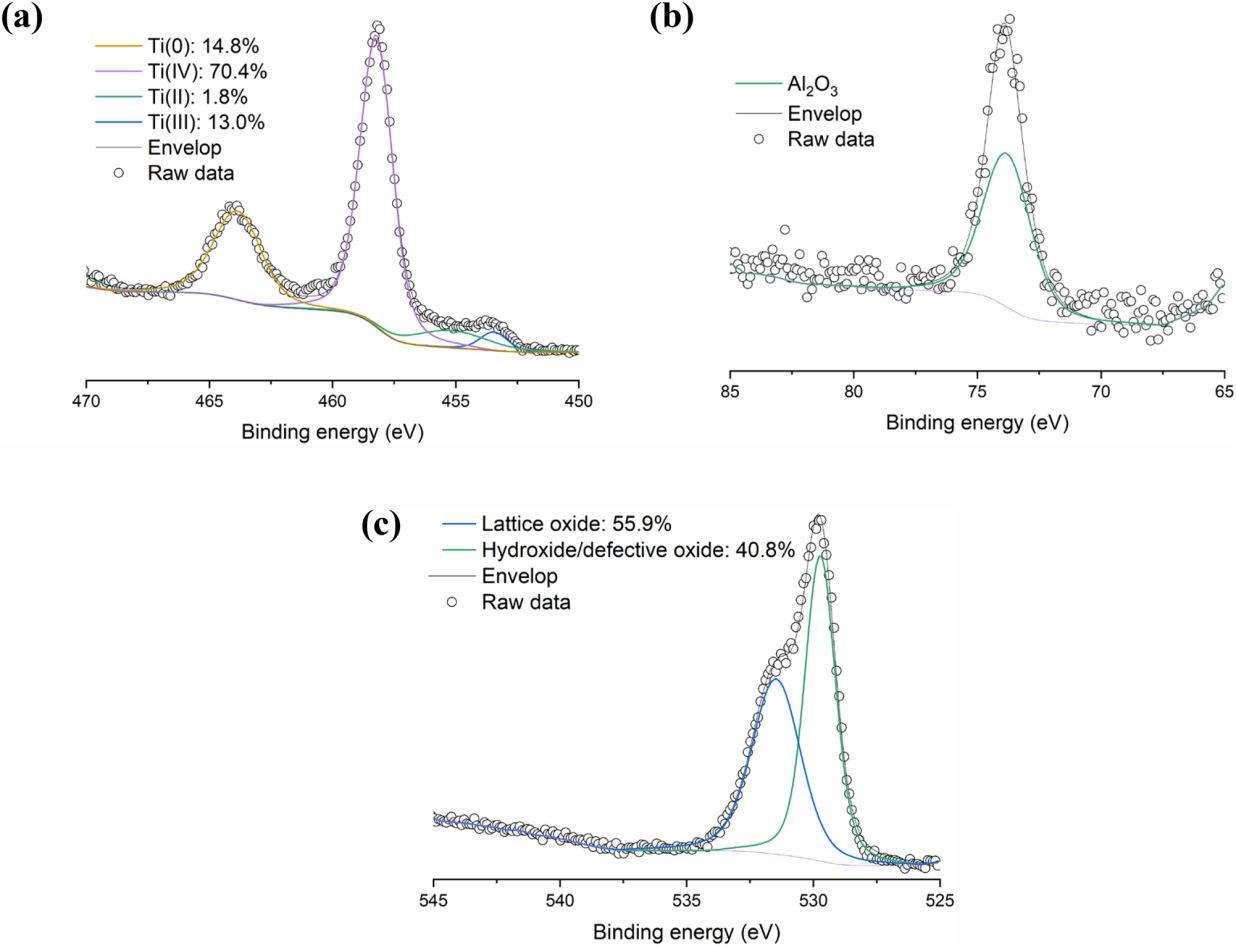
High-resolution (**a**) Ti 2p, (**b**) Al 2p, and (**c**) O 1s scans of Ti6Al4V powder.

As shown in Fig. 4, to determine the type of oxide at the surface, high-resolution scans of Ti 2p, Al 2p, and O 1s were obtained. In Fig. 4a is shown that the fitting of the Ti 2p peak is complicated due to the presence of spin–orbit split doublets with Ti 2p_3/2_ and Ti 2p_1/2_ components. The Ti 2p_3/2_ metal peaks were found at 453.9 eV and 454.1 eV for the powder. It was observed that these Ti 2p metal peaks alone are not sufficient to describe the Ti 2p spectrum of the sample. Therefore, it was concluded that other Ti species (such as oxides) are present as well. Adding Ti(II), Ti(III) and Ti(IV) peaks permitted to resolve the Ti 2p spectrum. The 2p_3/2_ peaks were found at 455.5 eV for Ti(II), at 457.2 eV for Ti(III), and 458.7 eV for Ti(IV). The presence of these different oxidation states, as well as their binding energies, agree with those reported by other researchers^13,15,18^. The Al 2p spectrum is illustrated in Fig. 4b and indicates that the Al species at the surface of the Ti6Al4V powder is in the form of Al^3+^, which may be attributed to Al_2_O_3_ or to interstitial/substitutional ions in the oxide matrix^16^. As seen from the high-resolution O 1s scan in Fig. 4c, most of these oxides are lattice oxides (55.9–57.0 at. %) while others are defective oxides (~ 40 at. %).

#### AlSi10Mg powder

**Figure 5:**
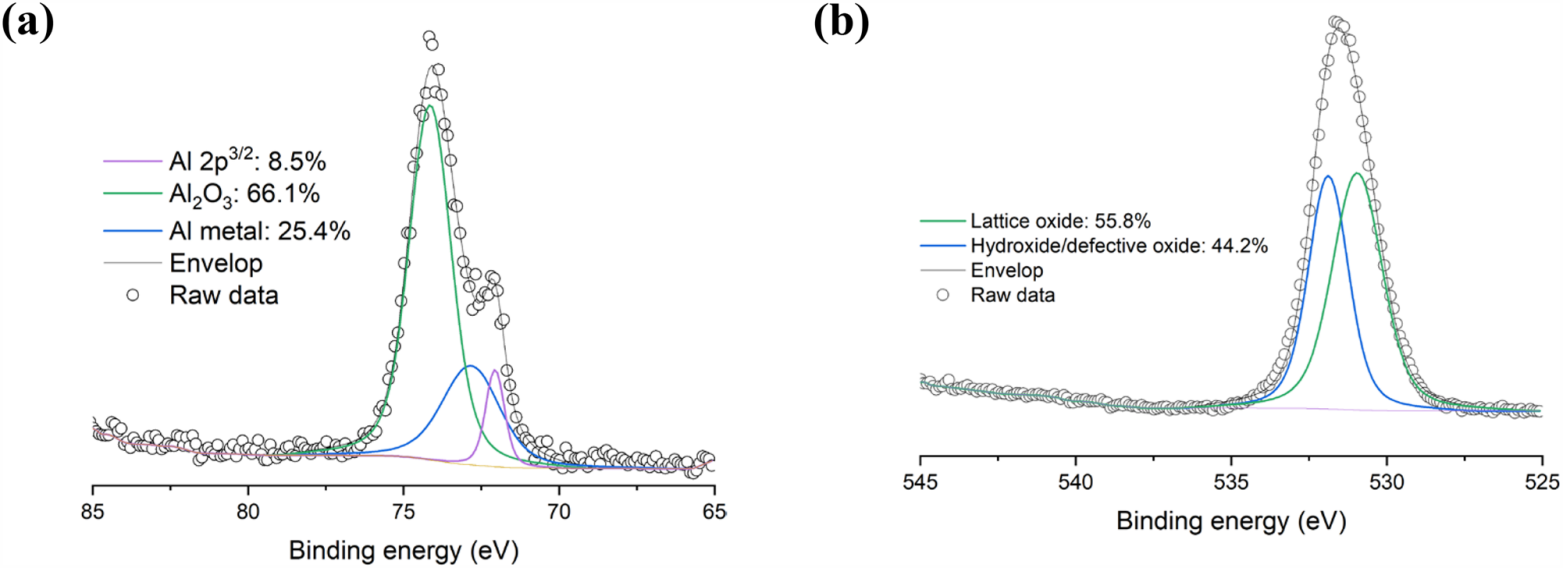
High-resolution (**a**) Al 2p and (**b**) O 1s scans of AlSi10Mg powder.

As presented in Table 4, the XPS elemental survey of the AlSi10Mg powder indicated the expected elements, namely, Al, Si, and Mg at the surface of the sample. To determine the type of species at the surface, Al 2p, and O 1s high-resolution scans were acquired. Figure 5a shows the high-resolution scan of Al 2p that was deconvoluted into its individual components and the dominant peak was found at 74.80 eV, which is assigned to Al_2_O_3_. Other peaks at 72.80 and 72.0 eV, corresponding to a possible hydrated aluminum specie such as AlOOH or Al(OH)_3_ and Al metal were also fitted. Figure 5b depicts the deconvolution of the O 1s spectrum into lattice oxide (at 530.2 eV) and hydroxide/defective oxide (at 531.9 eV) confirmed the presence of the aluminum species and yielded a relative composition of 55.8 and 44.2 at. %, respectively.

**Table 4:** Surface composition (in at. %) of AlSi10Mg powder from XPS elemental survey.

Sample	Al 2p	Si 2p	Mg 1s	C 1s	O 1s
AlSi10Mg	15.2	4.2	6.1	27.3	47.3

#### IN 738 powder

**Figure 6:**
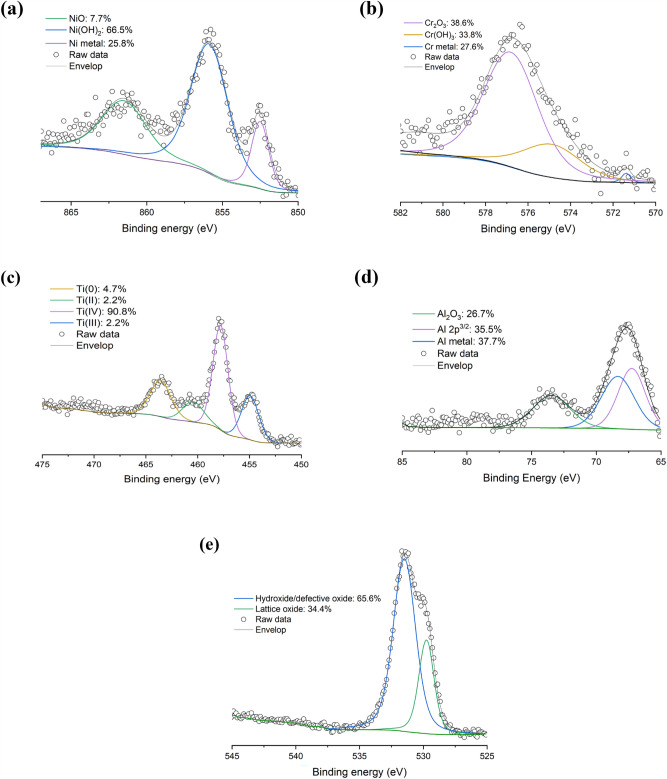
High-resolution (**a**) Ni 2p, (**b**) Cr 2p, (**c**) Ti 2p, (**d**) Al 2p, and (**e**) O 1 s scans of IN 738 powder.

Table 5 lists the XPS survey results of the elements found at the surface of the IN 738 powder. Ni, Cr, and O account to 3.7%, 3.0%, and 40.5%, respectively. Interestingly, 5.1 at. % of Ti and 4.8 at. % of Al are also present at the surface. Like other powders, C was detected as a contaminant at the surface of the sample. Unfortunately, after extensive research, no literature is available on XPS analysis of IN 738 at room temperature, preventing reliable comparisons. However, some studies at high temperature (> 900 °C) showed the presence of Ti and Al at the surface of samples^19,20^. Consequently, as shown in Fig. 6, the high-resolution scans of Ni 2p, Cr 2p, Ti 2p and Al 1s were conducted to determine the type of species at the surface.

**Table 5:** Surface composition (in at. %) of IN 738 powder from XPS elemental survey.

Sample	Ni 2p	Cr 2p	Ti 2p	Al 2p	C 1s	N 1s	O 1s
IN 738	3.7	3.0	5.1	4.8	39.7	3.2	40.5

As depicted in Fig. 6a, in the Ni 2p spectrum, only a small amount of NiO was detected. Most of the signal was attributed to Ni(OH)_2_ and Ni metal, which correspond to 66.5 at. % and 25.8 at. %, respectively. Figure 6b illustrates the deconvolution of the Cr 2p scan indicating the presence of Cr_2_O_3_ (main peak at 575.8 eV), but also Cr(OH)_3_ at 577.2 eV and Cr metal at 574.2 eV. On the other hand, Fig. 6c and d show the high-resolution scans of Ti 2p and Al 2p and they occur mainly as TiO_2_ and Al_2_O_3_, respectively. Also, Fig. 6e depicts the high-resolution O 1 s scan and confirms the presence of significant amounts of hydroxide at the surface. These results agree with those in the literature^19,20^ as some researchers have reported that the oxides present at the surface of IN 738 alloys at high-temperature are NiO, Cr_2_O_3_, TiO_2_, and Al_2_O_3_. Note that Ni(OH)_2_ was also observed depending on handling and storage conditions^21^.

#### SS 316L powder

Table 6 show the XPS elemental survey of the SS 316L sample. The presence of Fe, Cr, Mn, C, and O is observed at the surface of powder. Note that Mo, Ni and Si were not detected, which is consistent with the XPS analysis conducted by Yan et al.^22^.

**Table 6:** Surface composition (in at. %) of SS 316L powder from XPS elemental survey.

Sample	Fe 2p	Cr 2p	Mn 2p	C 1s	O 1s
SS 316L	10.2	2.6	3.5	33.8	50.0

**Figure 7:**
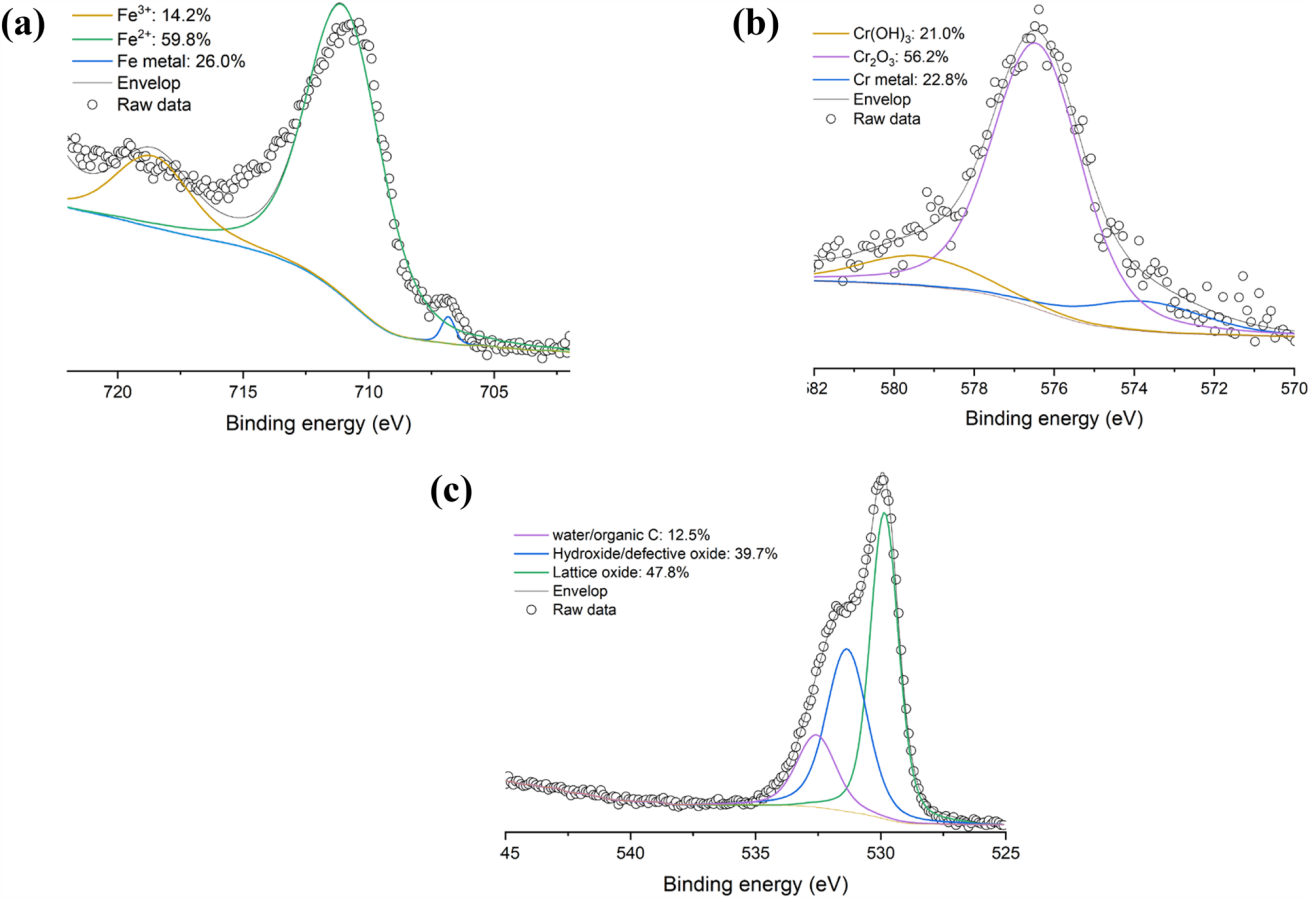
High-resolution XP spectra of (**a**) Fe 2p, (**b**) Cr 2p, and (**c**) O 1s of SS 316L powder.

The high-resolution spectra of Fe 2p and Cr 2p were acquired because Fe and Cr constitute the surface passivation layer of SS 316L^23^. As depicted in Fig. 7a, the deconvolution of the Fe 2p spectrum suggests that Fe has different oxidation states such as 0, 2+ and 3+, which have their main peaks fitted at 707.2 eV, 708.8 eV and 715.8 eV, respectively^13^. Figure 7b shows the Cr 2p spectrum, it can be fitted with a peak at 574.4 eV that can be attributed to Cr metal^24–26^, indicating that this specie is present at 22.8 at. % on the surface of the sample. The spectrum can also be fitted with Cr(OH)_3_ at 574.2 eV^25,26^ and multiple splitting peaks of Cr_2_O_3_ at 575.9 eV (for the main peak)^24^, respectively. In addition, as illustrated in Fig. 7c, the O 1s scan shows a peak at 532 eV which can be associated with adsorbed water, which may explain the presence of Cr(OH)_3_.

### Tribocharging behavior

#### CpTi powder

**Figure 8:**
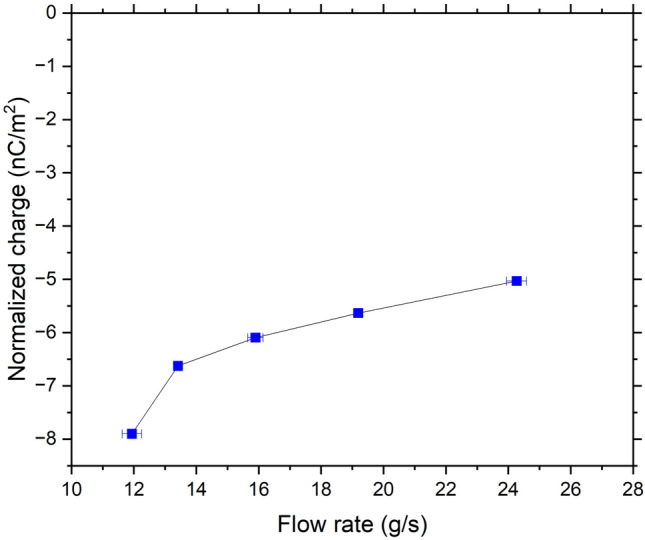
Measured accumulated charges of CpTi powder.

For the CpTi powder, we measured an initial charge of − 0.02 nC/g. Considering the SSA, this is equal to − 7.34 × 10^–3^ nC/m^2^, which is consistent with the value of − 1.70 × 10^–3^ nC/m^2^ reported by Kwetkus and Sattler^27^ for oxidized Ti powders. After flowing through the stainless-steel V-tube, the measured accumulated charges shown in Fig. 8 were in the range of − 5.03 to − 7.90 nC/m^2^ at the different flow rates tested. This could be due to the work function difference between the stainless steel and CpTi. In the XPS analysis of the CpTi powder (see section “CpTi powder”), the main oxide present at the surface is TiO_2_, which has an effective work function of 5–5.5 eV^27,28^, while the stainless steel (with Cr_2_O_3_ surface oxide) has an effective work function of 4.9–5.1 eV^29^. Both have insulating properties on the basis of their conductivity measurements, which also could be the reason why it is observe a charging dependence on flow rate^30^. Therefore, according to the theory of surface states, electron transfer must have occurred from the higher energy states of stainless steel (lower work function) to the lower energy state of TiO_2_ (higher work function), the latter gaining electrons (hence more negative) after contact, which is consistent with the measured final charges of the CpTi powder.

#### Ti6Al4V powder

**Figure 9:**
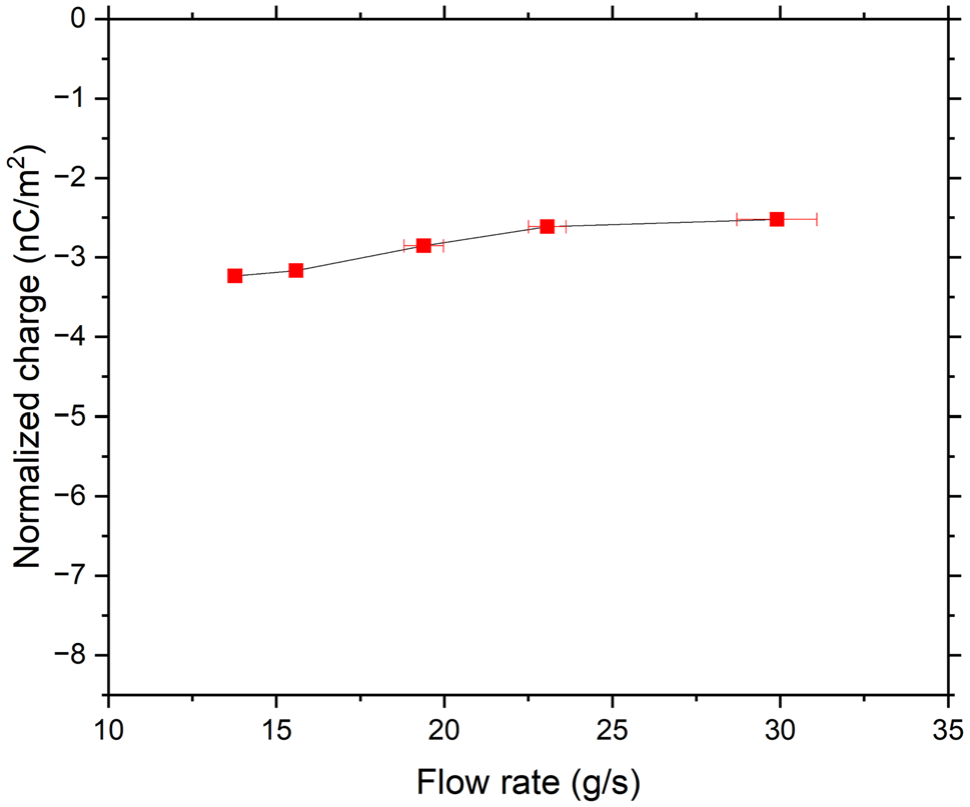
Measured accumulated charges of Ti6Al4V powder.

For the Ti6Al4V powder, the average initial charges obtained at the different flow rates vary between − 0.005 and − 0.008 nC/g, which correspond to − 4.9 × 10^–3^ C/m^2^ and − 7.4 × 10^–3^ C/m^2^, respectively, considering the SSA. These values are slightly lower than the − 0.02 nC/g initial charge we measured for the CpTi powder, owing to the presence of Al_2_O_3_ at the Ti6Al4V surface as indicated by XPS surface analysis. After flowing through the stainless-steel V-tube, the measured final accumulated charges shown in Fig. 9 are in the range of − 2.52 to − 3.23 nC/m^2^. As discussed in section “CpTi powder” about CpTi, since TiO_2_ has a higher work function than stainless steel (after flowing through the V-tube), Ti6Al4V acquires negative charges.

As seen, it is important to mention that even for powders fabricated with the same passivation process, such as in the case of CpTi and Ti6Al4V, the tribocharging response is significantly different. This is due to the different surface state on the surface of both CpTi and Ti6Al4V powder particles.

#### AlSi10Mg powder

**Figure 10:**
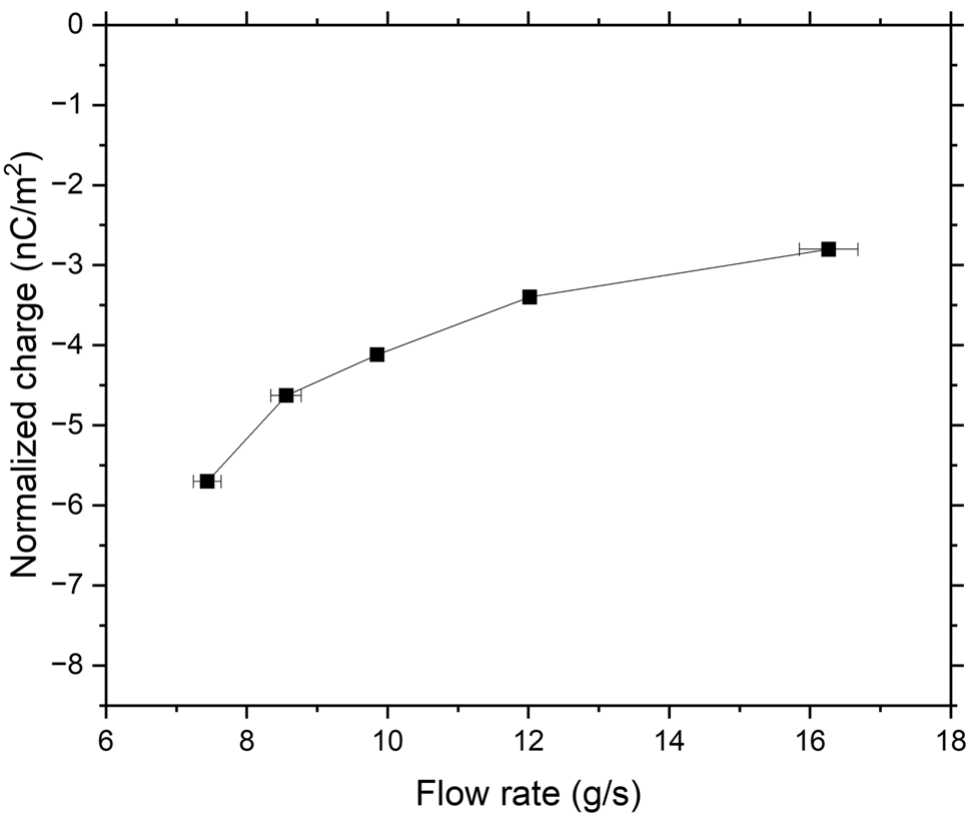
Measured accumulated charges of AlSi10Mg powder.

The average initial charge measured for the AlSi10Mg sample is − 0.009 nC/g. Considering the SSA, this value corresponds to − 1.1 × 10^–3^ C/m^2^, which is close to the charge density of − 0.9 × 10^–3^ C/m^2^ reported by Kwetkus and Sattler^27^ for Al–O. After flowing through the stainless-steel V-tube, the final accumulated charges depicted in Fig. 10 are in the range of − 2.80 to − 5.70 nC/m^2^. This shows, like our CpTi sample, a clear dependence on the flow rate in the V-tube. This can be ascribed to the insulation properties of Al_2_O_3_^30^, as stated in section “CpTi powder”. In addition, the AlSi10Mg sample became negatively charged mainly because Al_2_O_3_, which is the compound with highest concentration on the surface of the AlSi10Mg powder, has a higher work function than Cr_2_O_3_ on the surface of the V-tube.

#### IN 738 powder

**Figure 11:**
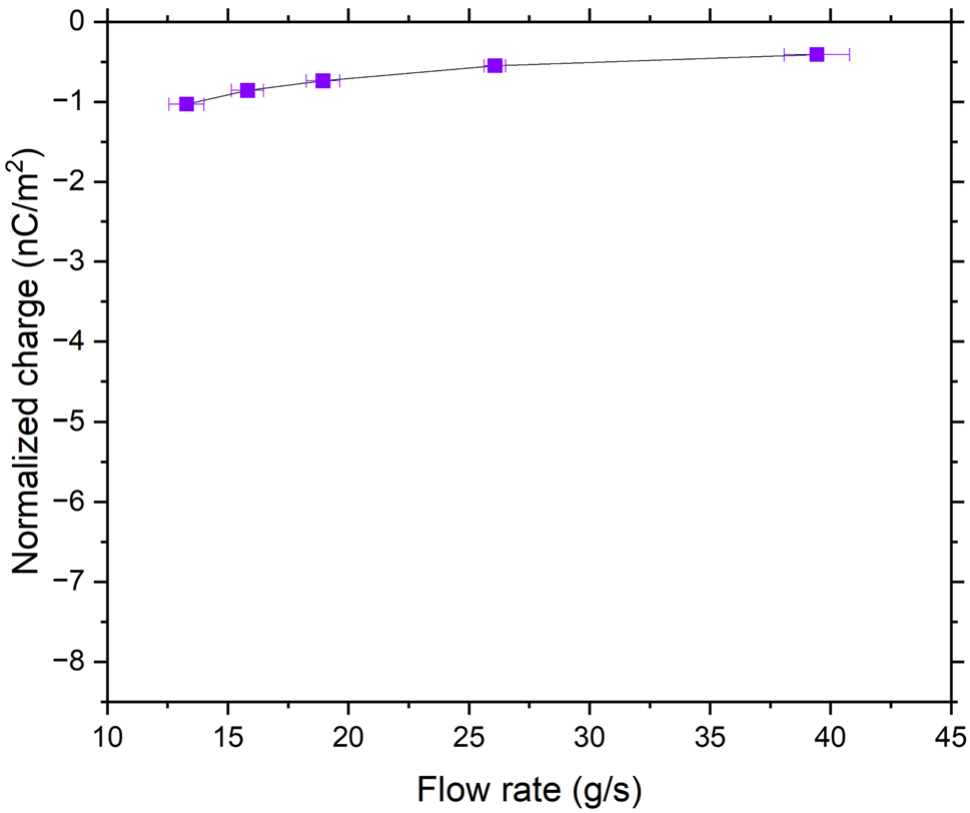
Measured accumulated charges of IN 738 powder.

The average initial charge measured for the IN 738 powder is − 0.001 nC/g, which corresponds to a charge density of − 3.8 × 10^–4^ C/m^2^, considering the SSA. As seen in Fig. 11, the accumulated charges range between − 0.41 and − 1.03 nC/m^2^, which is very small and relatively close to zero. This may be due to the presence of several surface oxides as shown in section “IN 738 powder” with different charging behavior leading to an overall charge close to zero. Moreover, the very small range of accumulated charges indicates that the charging behavior of the powder does not seem to be affected by the flow rate. Apart from the presence of contrasting oxides, it is believed that this behavior can be attributed to the presence of Ni(OH)_2_, detected at 66.5 at. % at the surface. Some polymorphs of Ni(OH)_2_ exist as layers intercalated with water molecules^31^. Due to the dipolar nature of water molecules, Ni(OH)_2_ has the ability to reorient its dipole in response to the movement of electrons brought about by triboelectrification^32^. This can be also explained by the ion transfer mechanism, which states that when two materials are in contact, mobile ions move between them. This is even enhanced with the presence of water at the surface^9^. In addition to ion transfer, surface charge relaxation through the interaction of accumulated ions with air could be another plausible explanation for this phenomenon^33^.

#### SS 316L powder

**Figure 12:**
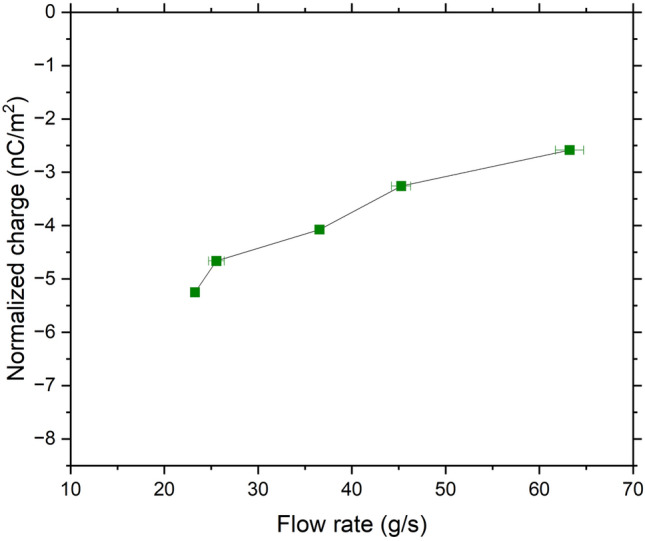
Measured accumulated charges of SS 316L powder.

For the SS 316L powder, the average initial charge measured is − 0.003 nC/g. Considering the SSA, this value corresponds to − 1.0 × 10^–3^ C/m^2^, which is close to the charge density of − 1.4 × 10^–3^ C/m^2^ reported by Kwetkus and Sattler^27^ for Cr–O. The final accumulated charge as a fuction of flow rate shown in Fig. 12 of the SS 316L powder is in the range of − 2.58 to − 5.25 nC/m^2^. This dependence is surprising since the stainless steel V-tube has the same type of surface oxide as the powder, suggesting that no charge could be created as the two materials have similar work functions. Several explanations can be proposed to explain the work function deviations observed. This includes spontaneous tribocharging caused by spot charging due to the similarity of the materials^34^, acquisition of ions from the atmosphere^34^, differences in crystallographic orientations of the materials^35^, surface roughness^35,36^, and presence of contaminants^37^ which can cause a variation of the electron density distribution at the surface^28,35^. All these factors can create slight work function deviations and thus charging even if the materials are composed of the same surface oxide^38^. In line with this, it is proposed that a different V-tube material could be used for SS 316L powder to better assess its tribocharging behavior.

## Discussion

**Figure 13:**
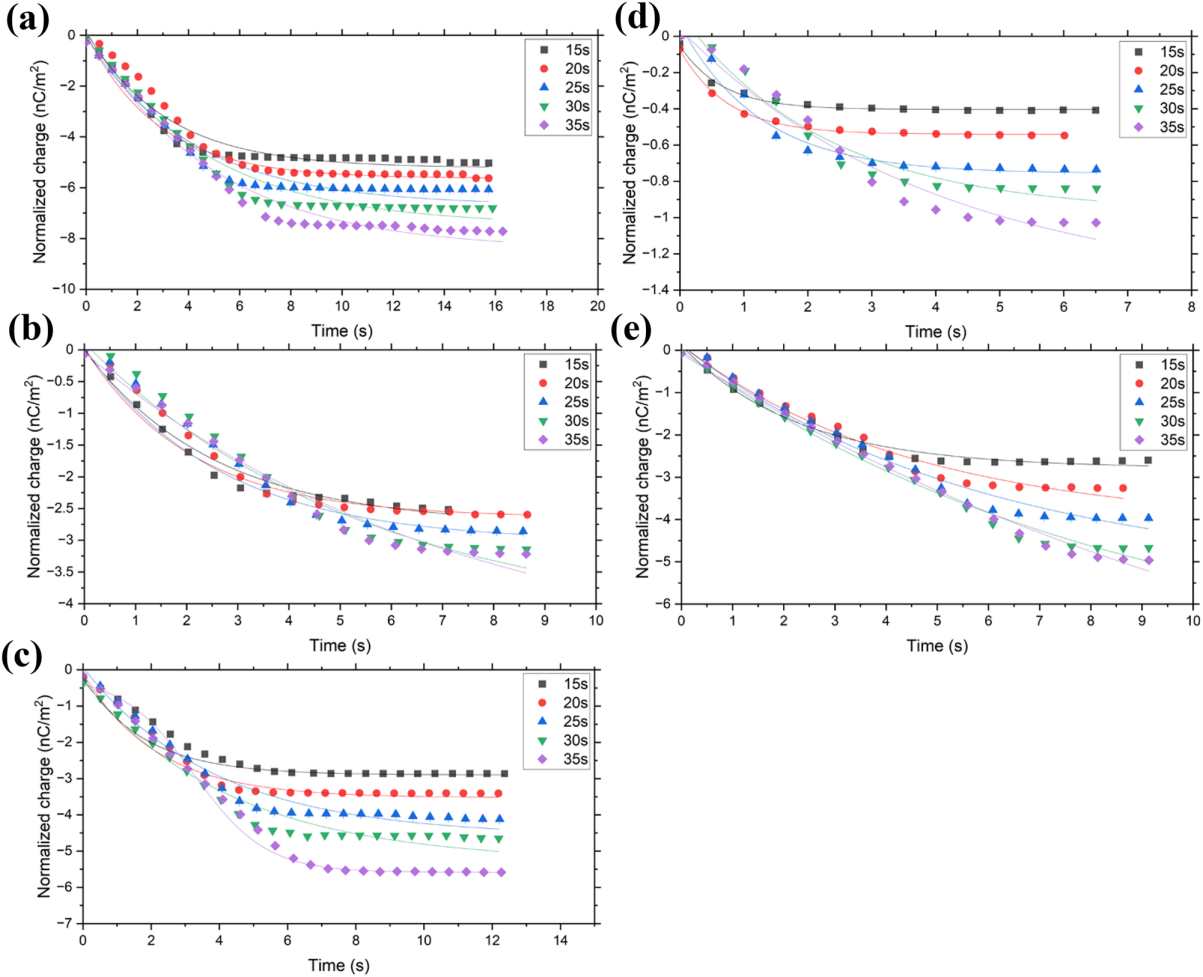
Model fitting of the charge vs time of (**a**) CpTi, (**b**) Ti6Al4V, (**c**) AlSi10Mg, (**d**) IN 738, and (**e**) SS 316L powders for different feeding times.

Different exponential models are known to fit charging curves^39^. In this work, the relaxation model initially employed was in the form of a stretched exponential. This model was first proposed by Kohlrausch^40^ in 1854 to describe the charge relaxation using a glass Leiden jar. Since then, the model has been widely used in dielectrics^41^. Here, Eq. (1) is similar to the charging model of Greason^42^:

1 $$Q\left( t \right) = Q_{f} \left( {1 - {\text{exp}}\left( { - \alpha t} \right)} \right),$$

where Q is the charge density at time *t*, *Q*_*f*_ is the final charge density, *α* is the charging rate, and *t* is the time. The charging rate *α* is equal to *1/τ*, where *τ* is the time constant. The above-mentioned relaxation model was modified since it is hypothesized that the charging rate is not constant but rather increases proportionally to *τ*^*β*^, where *β* is not the discharging rate but the stretch constant. The equation was then rewritten by Trachenko and Zaccone^2^ as a compressed exponential model described by Eq. (2) as:

2 $$Q\left( t \right) = Q_{f} \left( {1 - \exp \left[ { - \left( {\frac{t}{\tau }} \right)^{\beta } } \right]} \right),$$

where the compressed exponential *β* > 1. The compressed exponential model indicates that the charge relaxation is faster than that of the simple exponential model (where *β* = 1)^2^. Equation (2) was used to fit the respective tribocharging data (charge vs time) as shown in Fig. 13. Data fitting yielded averaged calculated *β* values of ~ 1.5, so this value was found to be independent on the system and hence set as constant. Interestingly, this value was used previously by other researchers to describe systems that exhibit “jammed” dynamics^43^. Examples of these systems are said to be structural glasses, colloidal gels, entangled polymers, and supercooled liquids, which experience jamming when motion of individual particles become restricted, causing their motion to slow down^44^. This behavior is also typical of granular materials^44^ as used in this work, and could give rise to an increase in the charging behavior.

The constants calculated, *Q*_*f*_ and *τ*, which correspond to the final charge density and the time constant, respectively, are presented in Tables 7, 8, 9, 10 and 11. The time constants *τ* are in the range 2.9–4.7 for CpTi, 1.7–3.5 for Ti6Al4V, 3.80–6.20 for AlSi10Mg, 1.1–1.9 for IN 738, and 2.0–4.6 for SS 316L. The time constant *τ*, which indicates the time it takes for the charge to reach a certain equilibrium, overlaps between CpTi, Ti6Al4V, AlSi10Mg and SS 316L, while it is significantly low for IN 738. This may have something to do with the presence of water on its surface, which acts as a sponge for the ion transfer, allowing it to reach equilibrium faster than other metal powders.

**Table 7:** Calculated constants from the data fitting of CpTi powder.

Feeding time (s)	Flow rate (g/s)	*Q*_*f*_ (nC/m^2^)	*τ* (s)	*α* (1/s)	*ß*	*R*^*2*^
15	24.3	− 4.96	3.60	0.72	1.45 ± 0.06	0.98
20	19.2	− 5.98	5.50	0.18	1.50 ± 0.02	0.99
25	15.9	− 6.15	4.70	0.21	1.46 ± 0.05	0.98
30	13.4	− 7.15	5.90	0.17	1.51 ± 0.02	0.98
35	11.9	− 7.94	6.50	0.15	1.55 ± 0.06	0.98

**Table 8:** Calculated constants from the data fitting of Ti6Al4V powder.

Feeding time (s)	Flow rate (g/s)	*Q*_*f*_ (nC/m^2^)	*τ* (s)	*α* (1/s)	*ß*	*R*^*2*^
15	29.9	− 2.63	3.20	0.31	1.36 ± 0.15	0.98
20	23.1	− 2.84	4.10	0.24	1.52 ± 0.00	0.99
25	19.4	− 3.44	5.70	0.18	1.46 ± 0.05	0.97
30	15.6	− 3.65	6.70	0.15	1.46 ± 0.06	0.96
35	13.8	− 3.65	5.80	0.17	1.51 ± 0.03	0.97

**Table 9:** Calculated constants from the data fitting of AlSi10Mg powder.

Feeding time (s)	Flow rate (g/s)	*Q*_*f*_ (nC/m^2^)	*τ* (s)	*α* (1/s)	*ß*	*R*^*2*^
15	16.27	− 2.98	3.80	0.26	1.56 ± 0.06	0.99
20	12.02	− 3.38	3.50	0.29	1.56 ± 0.06	0.97
25	9.85	− 4.29	4.90	0.20	1.50 ± 0.01	0.99
30	8.56	− 4.39	4.40	0.23	1.51 ± 0.00	0.98
35	7.44	− 6.20	6.20	0.16	1.50 ± 0.01	0.99

**Table 10:** Calculated constants from the data fitting of IN 738 powder.

Feeding time (s)	Flow rate (g/s)	*Q*_*f*_ (nC/m^2^)	*τ* (s)	*α* (1/s)	*ß*	*R*^*2*^
15	40.60	− 0.58	0.90	1.11	1.50 ± 0.09	0.99
20	26.50	− 0.58	0.90	1.11	1.55 ± 0.06	0.99
25	19.36	− 0.77	2.00	0.20	1.49 ± 0.01	0.99
30	16.41	− 0.96	3.10	0.32	1.49 ± 0.02	0.99
35	13.28	− 1.15	4.00	0.25	1.50 ± 0.08	0.99

**Table 11:** Calculated constants from the data fitting of SS 316L powder.

Feeding time (s)	Flow rate (g/s)	*Q*_*f*_ (nC/m^2^)	*τ* (s)	*α* (1/s)	*ß*	*R*^*2*^
15	63.2	− 2.60	3.40	0.29	1.59 ± 0.11	0.99
20	45.3	− 4.09	5.99	0.17	1.55 ± 0.06	0.98
25	36.6	− 4.83	7.10	0.14	1.45 ± 0.06	0.97
30	26.0	− 5.95	8.50	0.12	1.45 ± 0.06	0.98
35	23.3	− 6.32	9.30	0.11	1.40 ± 0.09	0.98

For a specific powder, it was also observed that the time constant *τ* increases proportionally with the magnitude of accumulated charge, while it is inversely proportional to the flow rate (i.e., the faster the flow rate, the smaller the negative charges gained). It is reasonable to presume that the slower flow rates lead to higher number of contacts, consequently leading to a higher accumulated charge, since more charges can be exchanged between the surfaces in contact^45^.

**Figure 14:**
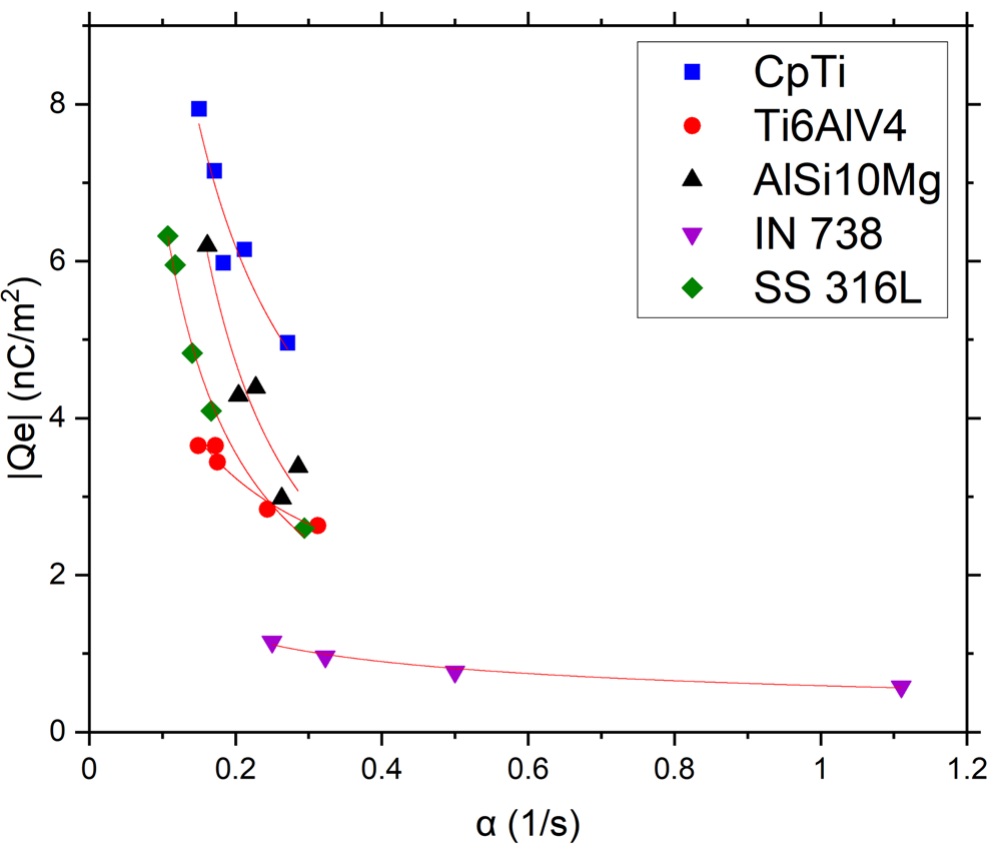
Model fitting of the normalized charge density as a function of the charging rate (α) for the powders.

The final step in this methodology links the surface charge accumulation and surface potential decay^46,47^. Considering that the maximum charge is a function of the charging rate, Eq. (3) can be used to determine the transfer efficiency that has to be a specific constant describing the relationship between powder surface composition, surface area and charging rate. Equation (3) lists:

3 $$Q_{f} = k\left( {\frac{dQ}{{dt}}} \right)^{n} = k\alpha^{n} ,$$

where *Q*_*f*_ is the absolute charge value, *k* is a constant, *dQ/dt* is the rate of charging with respect to time (*α*), and *n* is an exponent that varies depending on the specific triboelectric charging condition and material involved. Figure 14 represents this analysis using the constants presented in Tables 7, 8, 9, 10 and 11. The charging mechanism, *n*, is reported along with the powder characteristics in Table 12.

**Table 12:** Powders surface properties and calculated constant *n*.

*Sample*	*State*	*PSD, D*_*50*_	*Surface oxide*	*n*	*R*^*2*^
CpAl	As received	34	TiO_2_	0.75	0.88
Ti6Al4V	As received	36	TiO_2_, Al_2_O_3_	0.49	0.95
AlSi10Mg	As received	55	Al_2_O_3_	1.18	0.92
IN 738	As received	32	Ni(OH)_2_	0.42	0.98
SS 316L	As received	36	Cr_2_O_3_	0.89	0.89

Table 12 presents the start of the reference database correlating the surface composition with the tribocharging constants. The database will be augmented with different aging treatment (exposure to humidity, oxidation), to yield a comprehensive method permitting to indirectly determine the composition of powder surface scale. This simple method will be an interesting alternative to XPS or Auger techniques.

## Summary and conclusions

A new methodology for triboelectric charging was introduced. Few modifications with the current GranuCharge™ setup by incorporating an electronic flow rate measuring tool to assess tribocharging as a function of flow rate has been successful. The authors propose to measure the tribocharging behavior at several flow rates, instead of just one, because some flow rates may not indicate significant variation between two indistinguishable yet dissimilar powders, while other flow rates do. Minor variation of powder’s surface chemistry has proved to have affected the tribocharging response of the metal powders. Tribocharging behavior may not be the same even with powders having the same passivation oxide (e.g., CpTi and Ti6Al4V). The presence of negligible amounts of extra oxide (such as Al_2_O_3_) can cause considerable variation in powder tribocharging behavior. Even a small amount of water produced a significant deviation from the expected tribocharging behavior. Recognizing the degree of influence of moisture to the triboelectric behavior of AM powders is paramount to assess the degradation of powder quality. Hence, further studies on the interaction of moisture with AM powders and how it affects their tribocharging response are recommended. To better understand the triboelectric charging phenomena in AM powders, tribocharging models has also been proposed and was found to be in the form of a compressed exponential model with *β* = 1.5 and surface scales appear to be dependent on the value of the constant *n*, using the calculated values *Q*_*f*_, *τ* and *α* of different powder systems, which were used to compare their triboelectric charging behavior. The authors are intending to evaluate other powder systems than the ones studied in this work and expand the database of tribocharging constants for AM powders, which could open the gate to use the technique to identify surface states by the tribocharging constants knowledge, as an alternative of analytical techniques like XPS.

## Data Availability

The datasets generated and/or analysed during the current study are not publicly available due to the data also forms part of an ongoing study but are available from the corresponding author on reasonable request.
